# Real-World Application of an AI-Assisted Digital Workflow for Clinical Observational Data Collection in Dermatology Residency Programs: An Innovative Pilot Report From India

**DOI:** 10.7759/cureus.94688

**Published:** 2025-10-15

**Authors:** Naveen Manohar, Shruthi S Prasad

**Affiliations:** 1 Dermatology, The Oxford Medical College, Hospital & Research Centre, Bangalore, IND; 2 Dermatology, Indira Medical College and Hospitals, Pandur, IND

**Keywords:** artificial intelligence in medicine, data collection, dermatology residency curriculum, observational cross-sectional study, research methodology

## Abstract

Introduction: Residency research is a cornerstone of academic dermatology training, yet paper-based data collection remains labor-intensive, error-prone, and a major source of stress for trainees. Electronic data capture (EDC) platforms can mitigate these issues but are often costly and difficult to implement in resource-limited settings. We evaluated a low-cost, artificial intelligence (AI)-assisted workflow leveraging widely available Google Workspace tools to streamline data collection and enhance data integrity during dermatology residency research.

Methods: In this prospective, single-center simulation study, 100 hypothetical patient records with 41 variables each (total 4,100 fields) were independently entered using (i) conventional paper-based data collection with subsequent manual digitization, and (ii) a digital workflow using Google Forms, automated coding in Google Sheets, PDF generation, and real-time AI-assisted validation via ChatGPT. Mean entry time per record, error rates per field, and absolute risk reduction were calculated. Paired t-tests, McNemar’s test with continuity correction, exact binomial p-values, and Newcombe 95% confidence intervals were used for analysis.

Results: The AI-assisted workflow reduced mean entry time from 24.4 ± 1.5 minutes to 7.5 ± 1.1 minutes per record (mean paired difference=16.9 minutes, 95% CI: 16.61-17.19, p<0.001, Cohen’s d=11.66). Error rates decreased from 8.54% (350/4,100) to 2.39% (98/4,100), yielding an absolute risk reduction of 6.15 percentage points (95% CI: 4.82-7.47) and a 72% relative reduction in errors (McNemar’s χ²(1)=6.13, p=0.013; exact binomial p=0.0078). No cases demonstrated new errors unique to the digital workflow.

Conclusion: An AI-assisted, Google Workspace-based workflow significantly reduced both time and error rates in simulated dermatology research data collection. This approach is low-cost, rapidly deployable, and scalable to resource-constrained academic centers. Adoption of such workflows has the potential to improve research efficiency, enhance data integrity, and reduce resident stress, ultimately fostering a stronger research culture in dermatology training programs.

## Introduction

Evidence-based medicine relies on high-quality research, which in turn rests upon strong study designs, accurate data collection and analyses, and meaningful interpretations. Data collection and interpretation are time-consuming processes, especially in high-volume or resource-limited settings. Therefore, one of the keys to high-quality research output remains the ease of data collection and interpretation. In India, doctors may conduct research for the first time during their residency training. The current residency guidelines require completion of a thesis study during residency to be eligible for board certification [1]. However, conducting research during residency is difficult since it may be the first research study for residents, while they are simultaneously trying to learn a subject in depth and attend to patients [2]. The process may become so stressful that they get deterred from pursuing research studies in the future [3].

A good research study must include good strength of results, have a reproducible methodology, and maintain accurate data for others to analyze [4]. A study with a strong level of evidence requires time to gather and analyze data. While time may not be an obstacle for dedicated and well-funded teams, shortage of time is one of the obstacles for research during residency [3]. Therefore, if this process can be improved without compromising the quality of evidence, we can improve the research output of individuals as well as institutions. The quality of a study does not necessarily mean a large study population alone; its study design must be simple enough to replicate so that more evidence can be gathered on the topic. Lastly, data accuracy refers to the retention of the integrity of data while digitizing and analyzing it. Manual data entry is prone to errors, which can affect the accuracy and validity of the results. Therefore, attempts must be made to minimize data errors.

The typical thesis cycle includes the following steps: formulation, approval, paper data collection, digitization, cleaning of data, master chart, numerical coding of master chart, analyses, drafting manuscript, and submission. The formulation and approval steps are relatively simpler since both residents and their guides are involved actively in them; however, the subsequent stages are completed by the residents. Conventional thesis workflows rely on paper-based data collection followed by manual digitization, which is time-consuming, error-prone, and burdensome for residents. Therefore, in this study, we evaluated the feasibility and efficiency of a low-cost, AI-assisted digital workflow to streamline data collection, ensure accuracy, and provide real-time feedback for residents.

## Materials and methods

**Figure 1:**
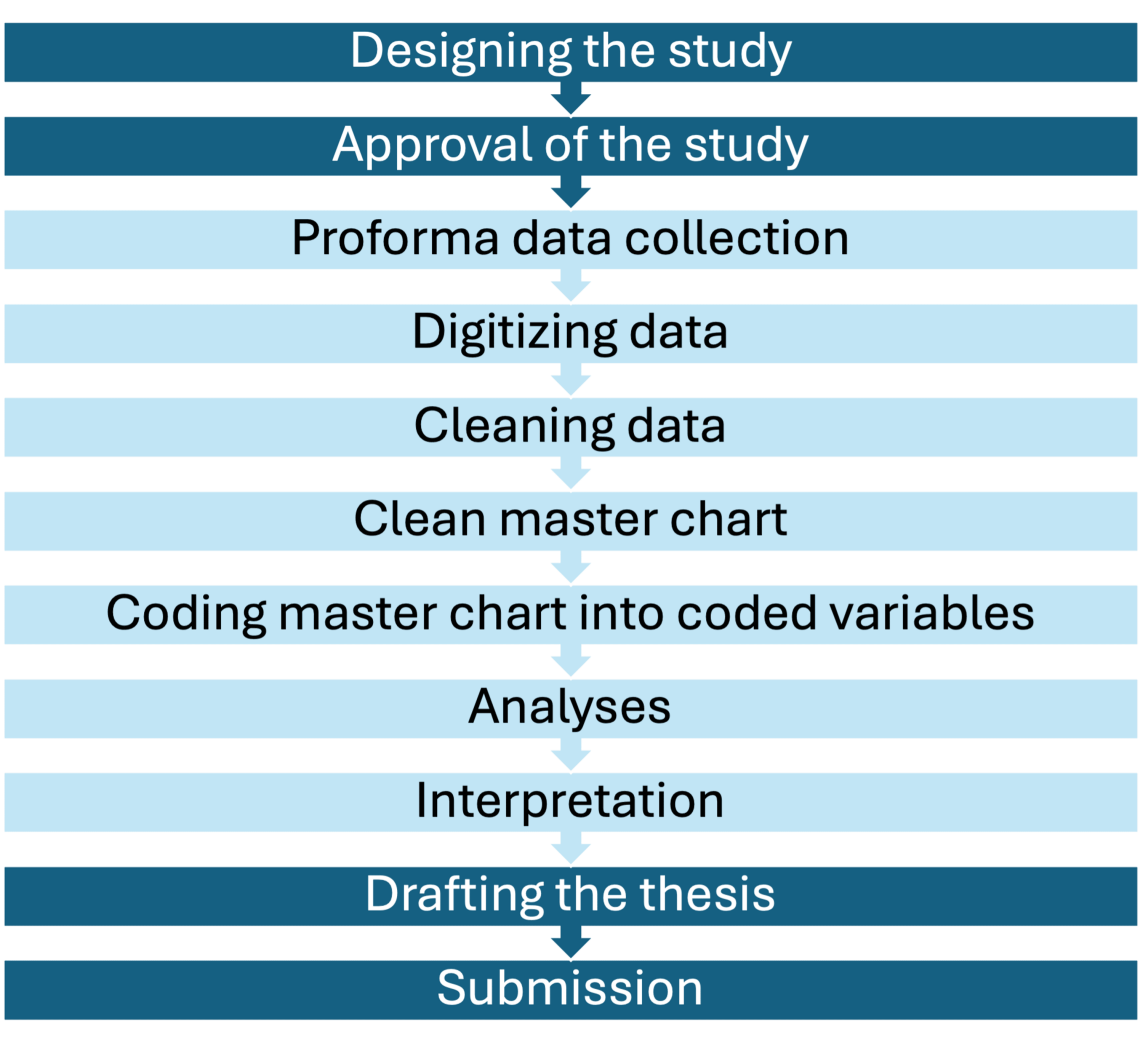
A flowchart depicting the typical steps involved in thesis completion by a dermatology resident over three years. We aimed to simplify the light-colored steps using the help of artificial intelligence (AI).

In this pilot feasibility study, we chose an observational, cross-sectional study design because data collection is the simplest with a single time-point of observation. Each observation is a row in the final master chart. We collected simulated patient responses in the study. The institutional ethics committee reviewed the pilot and confirmed that the use of simulated responses did not warrant formal approval. All participating residents provided informed consent. Figure 1 depicts the usual steps involved in a research thesis study and the steps we aimed to simplify.

Setup and tools

We used Microsoft Excel (Microsoft Inc., Redmond, VA, USA), ChatGPT (OpenAI, USA), and Google (Alphabet Inc., Mountain View, CA, USA) Forms, Sheets, Docs, and Apps Script, (hereafter called Forms, Sheets, Docs, and Apps Script, respectively) (Alphabet Inc., USA). First, we created a non-personal account on Google. In its Google Drive, we created a master folder for a sample project. Within that folder, we created the necessary Forms, Sheets, and Docs.

The end point of the data collection process is to have a clean master chart to run analyses. Therefore, we first designed a template master chart in Excel based on the questions in the data collection proforma. We uploaded this sheet to ChatGPT and prompted it to help us convert the sheet into questions of appropriate format in Forms. Responses entered in a Form can be stored in a Sheets table. Each response/patient constitutes a row in a table with the questions in the proforma acting as the column headings in the sheet.

A function in computer programming refers to a set of instructions. In its simplest form, a program also is a function or a collection of functions. A function is first defined and then can be called to execute the instructions in order. The structure and syntax of a function depends on the programming language used. A medical analogy to better understand functions would be to imagine a function named draw_blood, which includes the steps to draw blood. Once the function is understood, it can be then executed in sequence to accomplish the task. We used the help of AI to create functions that run in the background in Sheets to help us accomplish our goals.

Stage 1: refining the proforma

**Figure 2:**
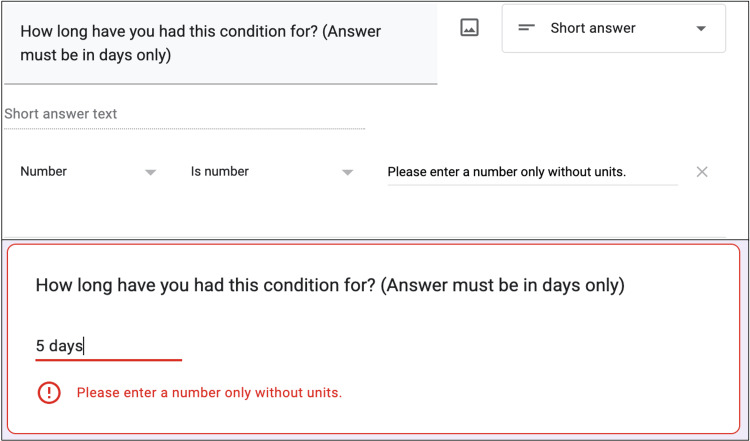
A screenshot showing the correct way to setup a question (above) along with validation (below). Textual input will not be accepted, thus ensuring that only numbers are entered.

This is the most critical step in the process of data collection. Each question must first be defined clearly in entirety along with instructions regarding acceptable answers. For example, the question “How long have you had this condition?” may seem acceptable, but the response may range from one hour to 10 years with varying units and accidental textual input. This lack of uniformity can result in textual and numerical responses that cannot be easily coded and analyzed. Therefore, the first step is to change the question to “How long have you had this condition? (answer in days only).” Consequently, the only responses can be numbers with days as the units, and they will not require additional text. Additionally, we used the validation option in Google Forms to ensure that only numbers are entered, and any text input can be rejected right away (Figure 2). No functions were used in this stage.

Stage 2: coding text into numbers

**Figure 3:**
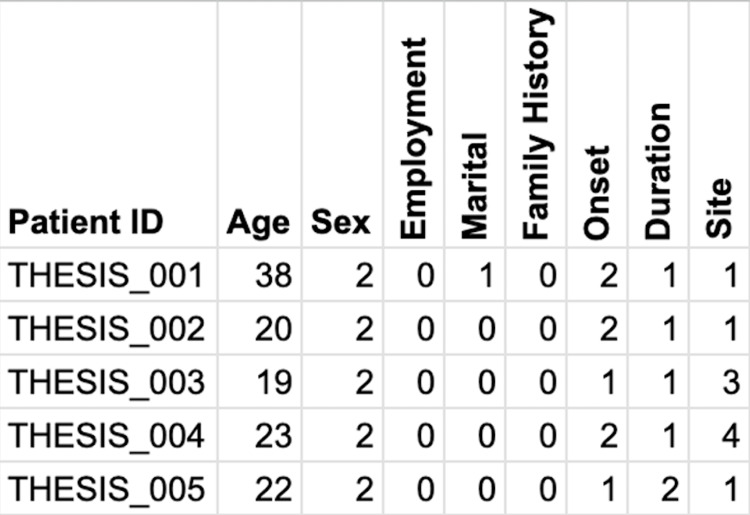
A screenshot showing coded information based on the original responses. Numerical values replaced textual responses using the following predefined dictionaries: sex = {1: female, 2: male}; employment = {0: unemployed; 1: employed}; marital status = {0: unmarried, 1: married}; family history = {0: no, 1: yes}; onset = {1, <6 months ago, 2, >6 months ago}; duration, the number represents the number of months; and site = {1: head, 2: head + trunk, 3: head + trunk + upper limbs, 4: head + trunk + both upper and lower limbs}.

For the next stage, in the Sheets where the responses were stored, we created an additional sheet named “Coded Responses.” The goal of this stage is to collect the responses from the “Main Responses” sheet, code them into numbers, and create a coded table in the “Coded Responses” sheet. In computer programming, a dictionary is a collection of key-value pairs, e.g., a dictionary of “sex” might include (1, female; 2, male; 3, others). Such dictionaries are used to convert text to numbers (female -> 1) or vice versa (1 -> female). Apps Script in Sheets (available under the menu “Extensions”) is based on JavaScript programming language and can be used to automate things in the background. We prompted ChatGPT the following: “The form responses are collected in a sheet. Please provide Apps Script code to create a new sheet that contains coded information based on the original sheet. In the new coded sheet, the first column must include a unique ID for each row only if it contains data. Dictionaries will be provided for each variable in the responses. This code should be triggered at each form response submission. The code should include ways to catch errors. In no way should the code alter the original submission.” The resulting code was then pasted into the script. We tested a few test submissions to ensure that this step worked. The function used in this stage was named generate_coded_sheet. At the end of this stage, each original response was recorded in the main sheet, and its corresponding coded numbers were recorded in the second sheet (Figure 3).

Stage 3: calculations

**Figure 4:**
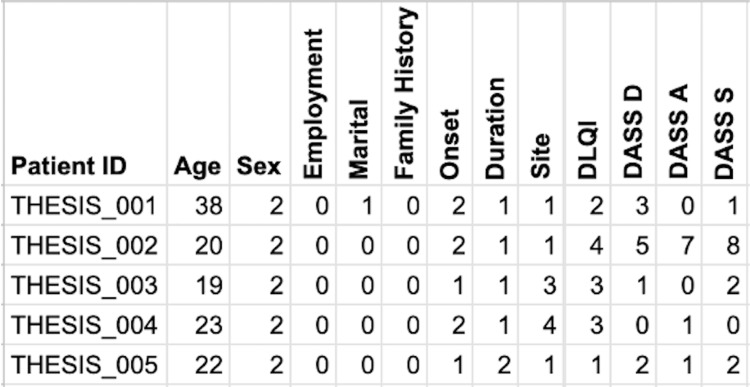
A screenshot of the coded database shows additional scores toward the right that were calculated by the system automatically. In addition to coding the textual responses into numbers (previous step), the system calculated DLQI and DASS-21 scores automatically and added them as columns. DLQI: Dermatology Life Quality Index; DASS: Depression, Anxiety, and Stress Scale; DASS D, depression subscale; DASS A, anxiety subscale; DASS S, stress subscale. Numerical values replaced textual responses using the following predefined dictionaries: sex = {1: female, 2: male}; employment = {0: unemployed; 1: employed}; marital status = {0: unmarried, 1: married}; family history = {0: no, 1: yes}; onset = {1, <6 months ago, 2, >6 months ago}; duration, the number represents the number of months; and site = {1: head, 2: head + trunk, 3: head + trunk + upper limbs, 4: head + trunk + both upper and lower limbs}. DASS21 scores are as follows: Depression: Normal: (0-9); Mild: (10-13);  Moderate: (14-20); Severe: (21-27); Extremely Severe: (28+). Anxiety: Normal: (0-7); Mild: (8-9); Moderate: (10-14); Severe: (15-19); Extremely Severe: (20+). Stress: Normal: (0-14); Mild: (15-18); Moderate: (19-25); Severe: (26-33); Extremely Severe: (34+).

Proformas include scoring scales, such as Dermatology Life Quality Index (DLQI) [5], Psoriasis Area Severity Index (PASI) score [6], and Depression, Anxiety, and Stress Scale-21 (DASS-21) [7]. We prompted ChatGPT with the following: “The form contains 10 questions regarding DLQI. Please provide code that can analyze the responses in the original sheet, calculate the DLQI score, and insert it into the respective row in the coded sheet. The original responses must not be altered.” The delivered code was tested for accuracy. Similar prompts were used for other scores. The functions used in stage included calculate_DLQI and calculate_DASS-21. At the end of this stage, additional columns were included in the second sheet with the respective calculated scores (Figure 4). This was a huge leap since now residents did not need to calculate the scores manually amidst their daily duties.

Step 4: digital proforma

**Figure 5:**
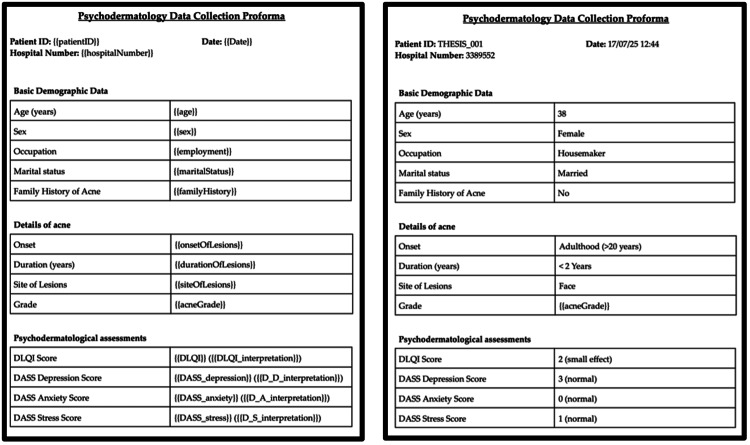
A screenshot showing the use of a template file (left) that is populated with the responses and calculated scores (right). DLQI: Dermatology Life Quality Index; DASS-21: Depression, Anxiety, and Stress Scale-21.

We created a template document in Docs for digital proformas (Figure 5). A template file is a document that includes a table with headings and placeholders within double flower brackets. We prompted ChatGPT with the following: “Google drive folder contains a template file. Please provide code to first duplicate this file so as to leave the original file untouched. In the copy made, the placeholders must be replaced with the corresponding information from the original and coded data. Then convert and save the document as a PDF in a folder named 'Digital Proformas' and delete the copy.” This process requires attention to detail since oversights can result in task failure, e.g., {{age}} and {{Age}} are two different items to the code. When a task failed, we reviewed the error and used ChatGPT to solve it. After a few tries, this step was being executed without errors (Figure 5). The function used in this stage was named generate_PDF. The result was a PDF file that could be printed for a physical backup.

Stage 5: interpretation

In Figure 5, we can see interpretation of scores next to the recorded scores. This was achieved by prompting ChatGPT the following: “In the function that generates the PDF, please provide code to also add the interpretation of the scores.” The resulting function was named interpret_score. Thus, we achieved recording the calculated scores and their interpretations in the PDF.

Step 6: communicate

**Figure 6:**
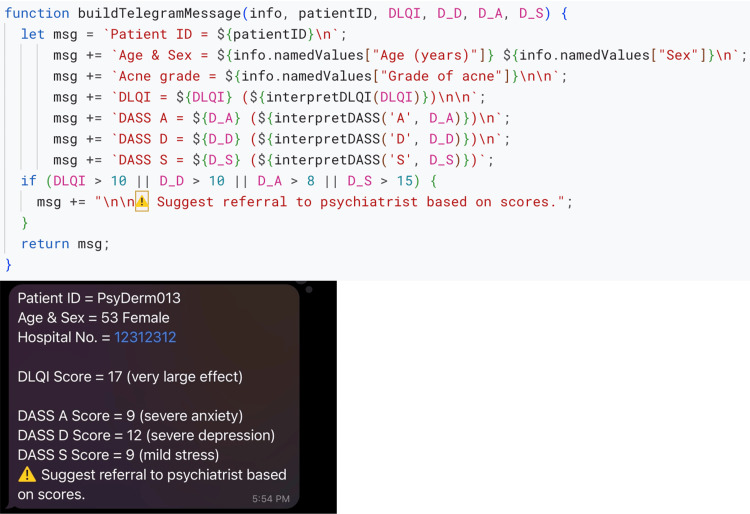
A function that includes a template Telegram message and populates it based on responses submitted (above). A screenshot of Telegram message received on a phone instantly (below). DLQI: Dermatology Life Quality Index; DASS-21: Depression, Anxiety, and Stress Scale-21.

After building this robust system, we wanted to relay a notification back to the responder or the resident. Telegram (Telegram Messenger Inc., Tortola, British Virgin Islands) is simpler to work with than WhatsApp (Meta Platforms Inc., Menlo Park, CA, USA). Therefore, we created a chatbot in Telegram on our personal phone and used ChatGPT to accomplish the following: “Please provide a script that can share the generated PDF instantly with the resident and thesis guide.” While we were running the script, we found it complex to work with PDF sharing. Therefore, we instead used a template text message in the script (Figure 6). Similar to the generation of the PDF, this message included placeholders that were replaced with the relevant information using a function named send_telegram_message, which used another function called buildTelegramMessage to use the relevant information into a text message (Figure 6). Additionally, if the scores were above a defined threshold, the text would also highlight the need to refer to a psychiatrist for further evaluation. Lastly, we used ChatGPT to include a few lines of code to email the PDF to the resident and the thesis guide for redundancy. This function was named send_email_with_PDF.

**Figure 7:**
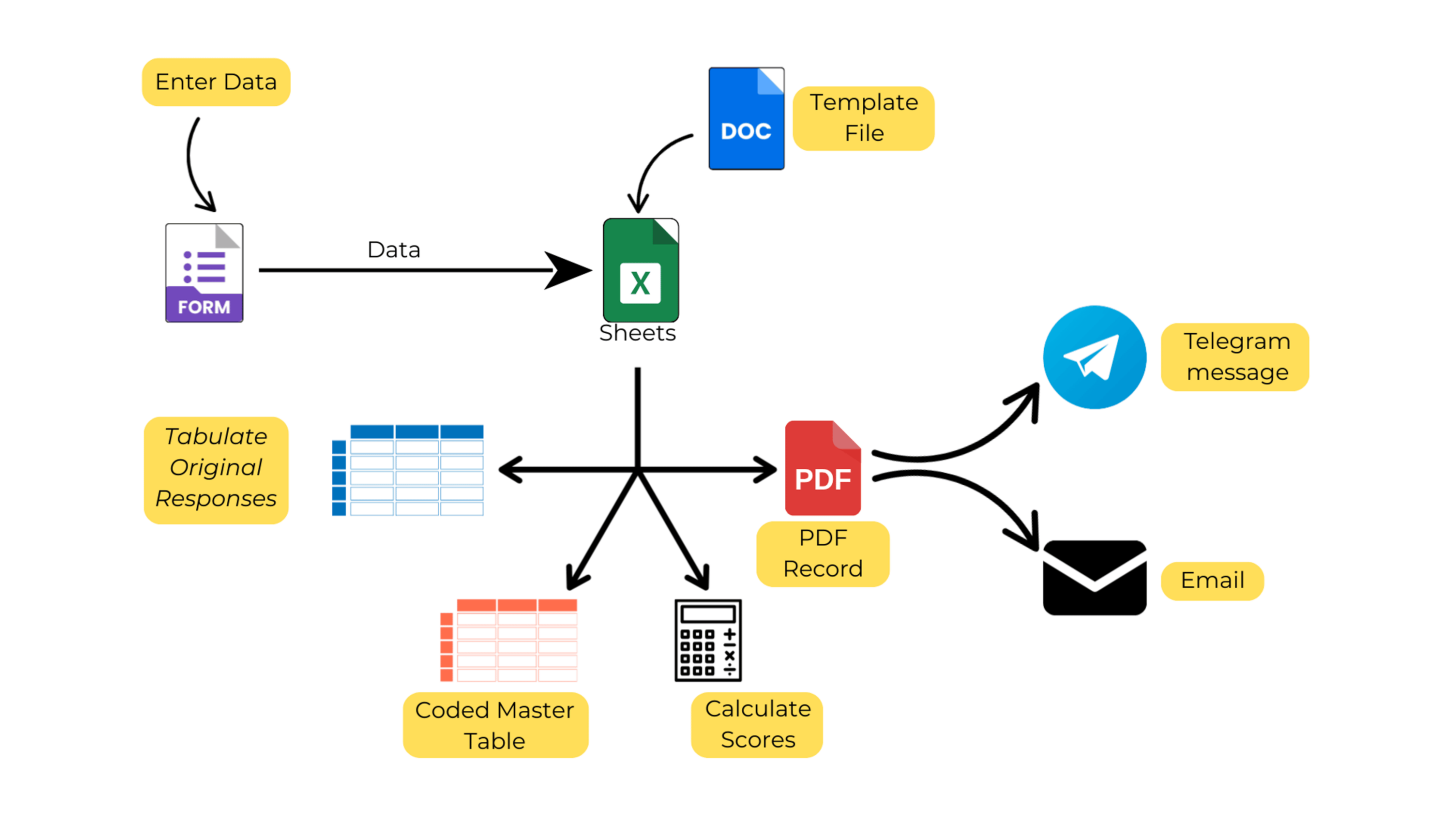
Visual representation of the workflow and its various arms.

We had now achieved a system in which upon administering proformas on a browser, coded and calculated databases were generated along with instant messages and emails to the residents and their guides. A visual representation of the workflow is presented in Figure 7. Table 1 summarizes the steps and functions used to achieve this workflow.

**Table 1:** A summary of the stages and functions used to achieve the digital workflow. DLQI: Dermatology Life Quality Index; DASS-21: Depression, Anxiety, and Stress Scale-21.

Stage	Goal	Function(s) Used
1	Refine proforma for objectivity and simplicity	-
2	Code responses into numbers	generate_coded_sheet
3	Calculate scores	calculate_DLQI calculate_DASS21
4	Prepare digital proforma	generate_PDF
5	Interpret scores	interpret_DLQI interpret_DASS21
6	Communicate via email and Telegram	send_telegram_message, send_email_with_PDF

Testing

For testing purposes, we provided a sample paper proforma with sections containing questions regarding demographic information (five questions), dermatological disorders (five questions), DLQI score (10 questions), and DASS-21 score (21 questions) to 10 residents. Each resident was required to collect 10 simulated responses from other residents acting as patients. Subsequently, they were instructed to enter the data in Sheets, calculate and interpret the respective scores, and code the responses into a master table. After a week, the exercise was repeated using the AI-assisted digital workflow. We measured the time taken for completion of the tasks and counted the number of incorrect digital entries, which included wrong scores and field-level mismatches.

Statistical analysis

Mean and standard deviations were calculated for the average time taken. Percentages were calculated for error rates. Paired *t*-test was used to compare the time required to collect data between the paper and digital approaches. Cohen’s d was used to quantify the effect size. Error rates were compared using McNemar’s test with continuity correction along with exact binomial p-values. The 95% confidence intervals for proportions and risk difference were computed using Wilson and Newcombe methods, respectively.

## Results

Time taken to collect data

Across 100 records, the mean time to complete data entry using the conventional paper-based method (followed by manual digitization) was 24.4 ± 1.5 minutes per record. Using the AI-assisted workflow, the mean time decreased to 7.5 ± 1.1 minutes per record. The mean paired difference was 16.9 minutes (95% CI: 16.61-17.19, t(99)=116.6, p<0.001), representing a 69% reduction in time per record. The effect size was very large (Cohen’s d=11.66).

Error rate

Across 4,100 total data fields (100 records × 41 fields), 350 errors were observed in the paper-based workflow (8.54%, 95% CI: 7.72-9.43%), compared with 98 errors in the AI-assisted workflow (2.39%, 95% CI: 1.97-2.90%). This represents an absolute risk reduction of 6.15 percentage points (95% CI: 4.82-7.47) and a relative reduction of 72%. Analysis of discordant pairs showed eight instances where an error was present only in the paper-based workflow and none where an error was unique to the AI-assisted workflow. McNemar’s test with continuity correction was statistically significant (χ²(1)=6.13, p=0.013), and the exact binomial McNemar test confirmed this finding (p=0.0078).

## Discussion

In this innovative technological report, we presented our low-cost approach to simplifying data collection in resident research using free digital tools. With the help of a few clicks or presses on a touchscreen, a real-time master chart can be updated on the go, along with coded parameters, interpretations of scores, and instant communication with residents and their guides. Residents were able to gather meaningful data rapidly and with fewer errors.

Conceptually, digital data collection for research is not a unique idea since there are Electronic Data Capture (EDC) platforms available for research data collection, such as REDCap [8], Medidata Rave EDC [9], Veeva Vault EDC (https://www.veeva.com), Oracle Clinical One EDC (https://www.appliedclinicaltrialsonline.com/view/oracle-unveils-clinical-one-0?utm_source=chatgpt.com), Castor EDC, Medrio, and Clinion EDC. However, they are primarily designed for large Phase I-IV trials with features that support global collaboration and automated data extraction. Additionally, they require subscriptions or fees along with a dedicated learning curve for each software. Setting up institutional servers for digital data collection for all residents and researchers may be limited by server costs, digital infrastructure costs, and developer or designer costs. These are not luxuries that most residents or teaching hospitals may be able to expend. Therefore, we aimed for the best utilization of free tools available at our disposal and use AI to build a robust system for data collection and preliminary analyses.

The extent to which this system can be implemented is limited by the user adoption and the learning curve. “Computer programming” or “coding” may sound foreign or intimidating; however, a code is simply a set of instructions that run to achieve a goal. That is precisely where AI can help doctors, especially in resource-limited settings. It can help a non-programmer convert an idea into a workable implementation. Caution dictates that we are running computer code without understanding the minute details. Since we do not know what we do not know, we may not identify errors or follow best practices of coding. This is not ideal for complex databases or sensitive data. Therefore, a critical step in this approach is to first understand safety practices while running code online (AI can help explain them!). For instance, we used non-personal accounts to test and run these codes. Additionally, we spent a lot of time asking AI tools to help us understand each step and how to safeguard it. Consequently, the total time taken to design the first working protocol for an observation study was 10 days. However, for another real study, we were able to replicate a customized workflow in two days.

An important implication of this system was that it relayed real-time scores, such as DLQI and DASS-21 scores. This is a clinically meaningful step forward since this system can help refer patients for further evaluation and counselling without costing any extra time or workload to the residents or the patients. One of the features we wanted was the ability to receive instant updates. While the creation of a clean database is a good milestone, we wanted residents to be able to see their work instantly on their phones, which accomplishes two things. One, residents can spend more time with patients since they are saving time during data collection. Two, a real-time text message with relevant information helps residents get involved with the research better. This workflow should be able to attract residents to gather more data for research since their data collection workload is decreased by approximately 60%-70%.

This innovative approach is not limited to research only. The basic principles can be applied to clinical practice as well. Real-time calculation of severity scores can hugely unburden practitioners from using questionnaires and mathematical formulae amidst consultations. There are several online calculators available for scoring systems. However, the accuracy and longevity of these calculators may not be assured. Therefore, a simple Telegram bot or a Google Form designed to calculate scores can elevate one’s clinical practice using evidence-based techniques. As one gets comfortable with coding, one can further explore complex relationships between data parameters or identify patterns.

Limitations

This study has several limitations. One, the workflow was tested using only 100 responses; a larger sample size is desirable. Two, sitting before a screen and working with code and troubleshooting may not appeal to everyone. Third, if one does not understand code, it can be dangerous to run unknown code on personal accounts. Therefore, the workflow here may not be generalizable to various study designs and applications. Consequently, it is advisable to use a non-personal account to run such projects. Fourth, data protection is paramount while using patient information on online platforms. Therefore, deidentified or anonymous data transmission should be preferred over identifiable data or more secure services with encryption should be preferred for sensitive data. Lastly, the responses of the AI tools may wary between agents or at different instances. Therefore, the workflow may not provide the same code, which limits its reproducibility. We combated this issue by maintaining detailed logs and notes to be able to retrace and pivot if needed.

## Conclusions

In this innovative pilot report, we used free online tools to build a low-cost, robust digital workflow for data collection and instant communication during residency research with the help of AI. We believe that AI can help non-technical persons, such as doctors, achieve some success in solving niche problems as demonstrated in this report. We intend to continue our work to other study designs and customizations to increase residents’ involvement with research.
